# Travel in the Time of COVID: A Review of International Travel Health in a Global Pandemic

**DOI:** 10.1007/s11908-022-00784-3

**Published:** 2022-08-04

**Authors:** Gerard T. Flaherty, Davidson H. Hamer, Lin H. Chen

**Affiliations:** 1 School of Medicine, University of Galway, Galway, Ireland; 2 School of Medicine, International Medical University, Kuala Lumpur, Malaysia; 3 Department of Global Health, Boston University School of Public Health, Boston, MA USA; 4 Section of Infectious Diseases, Boston University School of Medicine, Boston, MA USA; 5 Center for Emerging Infectious Disease Research and Policy, Boston University, Boston, MA USA; 6 National Emerging Infectious Disease Laboratory, Boston University, Boston, MA USA; 7 Division of Infectious Diseases and Travel Medicine, Mount Auburn Hospital, Cambridge, MA USA; 8 Harvard Medical School, Boston, MA USA

**Keywords:** SARS-CoV-2, Transmission, Transport, Testing, Quarantine, Travel medicine, Vaccine, Health passport, Sustainable travel

## Abstract

**Purpose of Review:**

This review critically considers the impact of the COVID-19 pandemic on global travel and the practice of travel medicine, highlights key innovations that have facilitated the resumption of travel, and anticipates how travel medicine providers should prepare for the future of international travel.

**Recent Findings:**

Since asymptomatic transmission of the virus was first recognized in March 2020, extensive efforts have been made to characterize the pattern and dynamics of SARS-CoV-2 transmission aboard commercial aircraft, cruise ships, rail and bus transport, and in mass gatherings and quarantine facilities. Despite the negative impact of further waves of COVID-19 driven by the more transmissible Omicron variant, rapid increases of international tourist arrivals are occurring and modeling anticipates further growth. Mitigation of spread requires an integrated approach that combines masking, physical distancing, improving ventilation, testing, and quarantine. Vaccines and therapeutics have played a significant role in reopening society and accelerating the resumption of travel and further therapeutic innovation is likely.

**Summary:**

COVID-19 is likely to persist as an endemic infection, and surveillance will assume an even more important role. The pandemic has provided an impetus to advance technology for telemedicine, to adopt mobile devices and GPS in contact tracing, and to apply digital applications in research. The future of travel medicine should continue to harness these novel platforms in the clinical, research, and educational arenas.

## Introduction

On March 11, 2020, the World Health Organization (WHO) declared COVID-19 a pandemic. As the world faced this unparalleled global health, social, and economic emergency, the travel and tourism industries were among the most affected sectors with airplanes on the ground, hotels closed, and travel restrictions put in place around the world. International tourist arrivals fell by 74% from 2019 to 2020, with an estimated 11-fold loss in international tourism receipts compared to the 2009 global economic crisis [1]. Past travel recovery periods from the 9/11 attack in the USA (2001), SARS (2003), and the global economic crisis (2009) depressed global travel levels for periods of between 11 and 19 months. Regional variations in the recovery intervals were significant in the Americas following the 9/11 attack with protracted recovery periods of up to 42 months observed. Despite the rapid gains in scientific discovery about SARS-CoV-2, its ability to mutate and become more transmissible have complicated the world’s recovery from the pandemic.

During this historic period, travel clinics closed and staff were furloughed or reassigned to COVID-19 response. Many recommendations were developed to provide guidance on travel during the pandemic [2, 3, 4••, 5, 6, 7•, 8••, 9]. Thoughtful reflections discussed the impact of the pandemic on travel and travel medicine, and many adaptations were made in response [4••, 10–12, 13•]. Given the resumption of travel, travel medicine providers and the travelling public should consider how this pandemic will influence the future of travel medicine.

This narrative review aims to describe some major aspects of the pandemic that affected travel, the optimization of elements of pre-travel advice that will be useful for the future, highlight game changers that helped to restart travel, key data and digital services that have altered travel medicine practice, and some travel health risks beyond COVID-19 that persist.

## Literature Search Strategy

Between March 5 and May 5, 2022, the authors searched MEDLINE, Embase, and Google Scholar databases using search terms relevant to COVID-19 (e.g., “covid,” “SARS-CoV-2,” “pandemic,” “transmission,” “vaccine”) combined with search terms relevant to international travel (e.g., “travel,” “international,” “borders,” “air travel”). The search was restricted to publications in English. Evidence from recent systematic reviews, meta-analyses, and clinical trials was prioritized. The bibliographies of retrieved articles were manually searched for additional sources not yielded by the primary search. The gray literature was consulted for relevant online reports from reputable international agencies involved in travel and tourism.

## Impact of COVID-19 Pandemic on International Travel

Following the turbulence experienced in 2020 and 2021, the travel and tourism industry has entered a steady though sluggish recovery phase. Despite a 4% increase in international tourist arrivals in 2021 (415 million), compared to 2020 as a whole (400 million), arrivals during the second year of the pandemic were still 72% lower than the immediate pre-pandemic year of 2019 [14]. The regions with the most robust recovery of travel volumes in 2021 were the Caribbean, Mediterranean Europe, and Central America, with further declines in arrivals observed in the Middle East and Asia–Pacific. Although there was a moderate rebound in the second half of 2021, owing to increased traveler confidence resulting from successful vaccination programs in developed countries, clearer travel protocols, and some loosening of travel restrictions [15], the pace of travel recovery remains cautious and uneven, with significant variation in mobility restrictions and vaccine uptake.

Tourism direct gross domestic product plummeted from a peak of US$ 3.5 trillion in 2019 to US$ 1.6 trillion in 2020, when many countries suspended all non-essential inbound and outbound international travel, before recovering to US$ 1.9 trillion in 2021 [14]. An increase in tourism expenditure reflected in average receipts per arrival in 2021 has been attributed to the impact of lockdown-related savings in a segment of the population, longer durations of stay, and higher transport and accommodation prices [14]. International arrivals are predicted to return to 2019 levels by 2024 at the earliest [14].

A greater public awareness of the contribution of air travel to global greenhouse gas emissions, coupled with a vocal flight shaming movement [14, 16], inflationary pressures, and recent conflict-induced surges in fossil fuel energy prices, have further slowed the recovery in international travel. The shutdown of Russian and Ukrainian airspace since February 2022 has affected travel within Europe and imposed increased costs on long-haul travel between Europe and the Middle East and Asia–Pacific regions owing to flight detours. Lifting of travel restrictions has been a leading factor in the recovery of international travel. Despite the negative impact of further waves of COVID-19 driven by the more transmissible Omicron variant in the first quarter of 2022, modeling by the United Nations World Tourism Organization (UNWTO) anticipates gradual growth in international tourist arrivals of up to 78% in 2022, compared to 2021 [14].

Novel consumer travel trends have been recognized, which include a preference for short-haul travel and domestic staycations, greater consciousness around sustainable approaches to travel, renewed interest in rural and nature-based tourism, and a tendency towards longer, but fewer and more expensive trips [17]. Anecdotally, a pattern of “revenge tourism” has emerged in recent months, whereby travelers have combined several bucket-list destinations into a single, more complex itinerary. The marked reduction in discretionary leisure travel throughout 2020 and much of 2021 also drew attention to an emerging phenomenon of social media influencer tourism, which deserves further research [18].

## Relevant Effects of COVID-19 Pandemic and Mitigation Strategies

The extreme measures adopted by national governments to limit the importation and domestic transmission of SARS-CoV-2 have divided public opinion, with some considering them too restrictive, non-evidence-based, or inconsistently applied across countries. The counterargument points to the significant additional mortality which would undoubtedly have occurred in the absence of societal lockdowns, social distancing, face mask mandates, and international travel bans. With the benefit of hindsight, it is prudent to reflect on some of the adverse collateral effects of stringent pandemic mitigation strategies, if important lessons are to be learned for future pandemics.

Harmful consequences have been attributed to national border closures, international travel restrictions, and mandatory traveler quarantine policies in relation to quality of life, well-being, and mental health [19•]. Concerns have been identified regarding equity, equality, and the global distribution of burdens arising from international travel measures and the impact of border closures on family reunification when relatives were trapped in a foreign country. Specific cohorts were disproportionately affected, such as students studying abroad [20], refugees [21], and cruise ship employees [22]. Mandatory hotel quarantine was generally perceived as detrimental to the mental health of travelers, although there were perceived economic benefits to host countries at a time of diminished hotel activity. Environmental benefits accrued from improved air quality resulting from reduced vehicular traffic during lockdowns [23]. Cities that usually experience mass tourism, such as Venice in Italy, reported improvements in the quality of life of their residents [24].

This pandemic has raised awareness of the mode of transmission of respiratory viruses and the beneficial effects of mask-wearing in crowded indoor settings such as aboard commercial aircraft. There has also been a revolution in hand hygiene practices and widespread adoption of rapid antigen self-testing to guide social behavior domestically and in travel settings [25, 26]. The long-term benefits of travel-related infection prevention remain to be fully appreciated, but there are indications that pandemic control measures were responsible for a decline in the incidence of influenza and other respiratory viruses [27, 28], gastroenteritis [29], sexually transmitted infections [30], and dengue [31] in certain countries. Other collateral effects of the pandemic have included delays in the diagnosis of severe malaria [32], interruptions to national childhood immunization schedules [33], changing patterns of sex tourism [34], and flexible work practices, which have given rise to novel entities in travel medicine such as digital nomads and so-called workcations.

## Travel-Related Transmission of COVID-19

The role of international transportation in facilitating the spread of SARS-CoV-2 has been extensively investigated. Since pre-symptomatic transmission of the virus was first recognized in March 2020 [35], efforts have been made to characterize the pattern and dynamics of transmission aboard commercial aircraft [36], cruise ships [37], rail and bus transport, and in mass gatherings and quarantine facilities (Table 1). Whole-genome sequencing has contributed dramatically to understanding the spread of SARS-CoV-2 among aircraft passengers [38, 39]. An engineering simulation study from Hong Kong determined that front passengers exposed to a passenger coughing in the seat behind them had a four-fold greater infection risk than that of other passengers [40]. The risk of inhaling infected droplets from a talking passenger was broadly the same for nearby passengers. Wastewater-based epidemiology has emerged as a valuable tool for the surveillance of SARS-CoV-2 RNA and has been successfully applied in the setting of commercial aircraft and cruise ship sanitation systems as well as at mass gatherings including the Tokyo 2020 Olympic and Paralympic Games [41, 42].

**Table 1:** Examples of travel-associated transmission of COVID-19: cruise ship, air travel, quarantine or isolation facilities, other transport/group travel, and mass gatherings

**Arrival date/incident date**	**Origin of cruise/flight/other transport/participants**	**Incident location**	**Number of cases**	**Comments**	**References**
**Cruise ships**					
January–February 2020	Japan	Hong Kong, Japan	Diamond Princess: 712 cases (554 of 2666 guests, 152 of 1045 crew), 9 deaths	Of 437 Americans and their travel companions on the ship, 114 (26%) were SARS-CoV-2-positive. Attack rate was 18% in those without infected cabinmates vs. 63% with asymptomatic infected cabinmate vs. 81% with symptomatic infected cabinmate. Estimated infection rate was 79% if no intervention had been implemented	[43–45]
February–March 2020	USA	USA	Grand Princess 2 voyages: 123 cases (among 2422 guests and 1111 crew) and 5 deaths	Only 30% of guests and crew were tested. Of 469 persons with available test results, 78 (16.6%) were SARS-CoV-2-positive	[43]
March 2020	Australia	Australia	Ruby Princess: 907 primary cases (605 of 2647 guests and 202 of 1151 crew), 29 deaths	120 people on board the Ruby Princess met the case definition for COVID-19 at the time of disembarkment; in April 2020, the outbreak was linked to 13% of all COVID-19 cases in Australia	[37]
July–August 2020	Norway	Norway	MS Roald Amundsen: 42 cases among 167 crewmembers and 28 cases among 391 passengers (attack rates 25.2% and 7.2%, respectively)	Outbreaks of lineage B.1.36 occurred on 2 1-week voyages, from Tromsø around the Svalbard archipelago	[46]
**Air travel and quarantine isolation facilities**					
March 2020	UK	Vietnam	1 index case, followed by 16 cases among 201 passengers and 16 crew	Flight VN54: Among 16 cases, 12 (75%) were passengers in business class along with the only symptomatic case (attack rate 62%). Seating proximity had increased infection risk (risk ratio 7.3)	[47]
March 2020	USA	Hong Kong	4 cases (2 passengers among 294 passengers, and 2 crew)	The near full-length viral genomes from the 4 cases were 100% identical, were phylogenetically grouped to clade G, and were distinct from 189 other Hong Kong viral sequences collected during January–May	[48]
March 2020	Australia	Australia	29 cases PCR-confirmed (among 241 passengers on board)	6 initial cases identified to be on flight led to PCR confirmation of 18 primary cases: most had disembarked from cruise ships (Ovation of the Seas; Ruby Princess) before the Sydney-Perth flight, and one passenger had travelled from the USA, and 11 secondary cases. All WGS available were A2-RP strain	[39]
September 2020	India	New Zealand	Index cases tested positive in MIQ @3 days after flight, followed by sequential identification of 7 additional cases in MIQ and in the community	A chain of transmission without direct person-to-person contact by aerosol within MIQ; transmission in-flight, and within households; WGS analysis helped to identify probable direction of transmission between cases	[49]
September–October 2020	UAE	New Zealand	7 positive PCRs among 86 passengers on a flight from Dubai, UAE	Flight EK448: cases originated from Switzerland (2), Ukraine, Ireland, India, South Africa (2) before layover in Dubai; 5 had negative pre-departure PCR. All 7 SARS-CoV-2 genomes were genetically identical, except for a single mutation in 1 sample	[50•]
December 2020	International	Ireland	165 cases on 134 flights; 40% symptomatic on board	National study of SARS-CoV-2 on 2098 inbound international flights and estimated 135,900 passengers in December 2020; secondary attack rate of 7.0%, higher on flights ≥ 5 h	[51]
April 2021	India	Hong Kong	59 PCR-confirmed cases among 146 passengers; 20% symptomatic	Delhi-Hong Kong flight: 5 positive upon arrival. 7 were estimated to be infected prior to travel, 41 infected during transit, 11 infected in quarantine. WGS detected 3 variants Kappa, Alpha, and Delta	[52]
April 2021	India	Australia	47 cases aboard 2 flights carrying 345 passengers; 14% of arrival cases symptomatic	Based on analysis of SARS-CoV-2 genomic clusters, transmission occurred despite mandatory mask wearing and pre-departure testing. Pre-departure quarantine and enhanced pre-departure testing were implemented for subsequent flights	[53•]
April–June 2021	International	Spain	196 PCR-confirmed among 45,211 travelers initially tested by rapid antigen on arrival to Madrid international airport	Most cases came from Colombia (114), followed by the Dominican Republic (30) and Peru (12). WGS identified B.1.621 (Mu) to be the most frequently occurring variant, but others were also found, including Alpha, Beta, Gamma, and Delta. This showed potentially infectious passengers on board but did not trace their contacts to identify in-flight transmission	[54]
July 2021	Philippines, United Arab Emirates	New Zealand	Traveler A arrived from the Philippines and traveler E from a 5-person travel group (BCDEF) from UAE tested positive. Travelers B, C, and D subsequently tested positive; viral sequences matched A	In the MIF, traveler A and group BCDEF occupied rooms > 2 m apart across a hall and never had direct contact	[55]
**Other transport or group travel**					
January 2020	China	China (Beijing ex Wuhan)	A family of 3 travelled by train from Beijing to Wuhan to visit the younger son. One parent developed respiratory symptoms and COVID-19 was confirmed; subsequently, the other family members were confirmed	The younger son had been living in Wuhan since August 2019. The father travelled to Wuhan on January 4, 2020, and the mother and the elder son went to Wuhan on January 18, 2020. On January 20, they took the 4-h train to return to Beijing without wearing masks. Once they arrived in Beijing, they drove their own car back home and were isolated at home. The mother developed respiratory symptoms on January 23	[56]
August 2021	Israel	Iceland	Among 25 travelers on a 12-day tour (96% were fully vaccinated with 2 doses of BNT162b2), 21 became PCR-positive pre-departure for Israel or upon landing; attack rate 84%	All 25 travelers were PCR negative pre-travel, 15 (60%) tested PCR-positive pre-departure from Reykjavik, and 6 more tested positive upon landing. All cases were mild and none was hospitalized. The tour had a local bus driver (vaccinated), dedicated bus, with little indoor contacts with locals or other travelers	[57]
October 2021	Japan	Japan	19 cases identified (18 participants and 1 bus staff), the index case and majority of cases were on bus 1 (18/19 [95%], attack rate 44%), and one participant case on bus 4 (1/19 [5%], AR 3%)	158 persons (146 participants and 12 staff) participated in a 4-day tour on four buses visited 11 tourist sites around Hokkaido, Japan, in October 2020: 41 for bus 1, 39 for bus 2, 40 for bus 3, and 38 for bus 4	[58]
May 2021	International	Nepal	14 COVID-19-positive Everest travellers presented to CIWEC in 2021	5 had 2-dose vaccination with ChAdOx1 nCoV-19 (*n* = 2), Sinopharm (*n* = 1), Sputnik V (*n* = 1), and mRNA-1273 (*n* = 1), and one had single dose of ChAdOx1 nCoV-19. Among these, 3 required hospital admission and 3 were treated as outpatients. No death was reported	[59]
**Mass gatherings and superspreading events**					
February–March 2020	France	Switzerland	Phylogenetic analysis of positive SARS-CoV-2 tests determined the origin of B.1-C15324T to mid-February in the trinational region around Basel. Genome analysis of multiple early cases identified attendance at a religious mass gathering event in Alsace, France	The outbreak in Basel was dominated by lineage B.1 (83.6%), detected in early March. Within B.1, the majority of samples fall within a clade including 157 identical sequences at the root of the “Basel cluster,” some of which were traced to regional spreading events. A mass gathering event was the predominant initial source of cases	[60]
February–March 2020	USA	USA	An international business conference with one COVID-19-infected international attendee led to domestic and international spread, and sustained community transmission, including outbreaks in homeless and other higher-risk communities, resulting in > 300,000 cases	Genomic analysis of 772 SARS-CoV-2 sample identified > 120 introductions of SARS-CoV-2 into the Boston area, but only a few led to most local transmission: 29% of the introductions were responsible for 85% of the cases, including the international business conference. Most of the introductions occurred in March and early April, primarily from elsewhere in North America and from Europe	[61]
March 2020	Jordan	Jordan	An index patient at a wedding was linked to 85 cases that were confirmed in the 4 weeks following the event	About 360 persons attended a 2-h wedding ceremony and party at an indoor venue and were exposed to the index patient, the bride’s father. The wedding was linked to 85 subsequent positives where 76 (89.4%) attended the wedding, and 9 (10.6%) were close contacts of confirmed cases from the wedding	[62]
January–April 2021	India	India	Daily COVID-19 cases increased from 37 to 144 (276%) in Haridwar. In Uttarakhand state, daily cases increased from 138 to 480 (236%) and in India, from 45,600 to 92,754 (92%) during this MG	Kumbh Mela mass gathering (MG) took place from 14 January 2021 to 29 April 2021 at Haridwar, the capital city of the state of Uttarakhand, India. The estimated attendance was ~ 10–20 million people between 1 April 2021 and 30 April 2021	[63]
June–July 2021	International	Netherlands, Denmark, Scotland, England, Germany, Italy, Russia, Spain	An increase in COVID-19 incidence per 100,000 population from the start of Euro2020 was observed across 7 of 11 host cities/regions: Netherlands (1629%), Denmark (210%), Scotland (57%), England (382%), Germany (9%), Italy (104%), Russia (196%) and Spain (135%)	COVID-19 cases across declined in May 2021 in Europe as vaccination rates were on the rise. As Euro2020 initiated in June 2021, an increase in COVID-19 cases was observed along with Delta variant, across all countries in Europe. Except for Budapest which had no increase, all host cities/regions exhibited an increase between the start of the matches and 15 days after the last match played in the city/region. Munich-Germany experienced a minimal increase	[64]
November 2021	USA	USA	Overall 119 (2.6%) persons from 16 jurisdictions were positive from among 4560 SARS-CoV-2 tests	An indoor convention in New York City was attended by about 53,000 vaccinated persons from 52 US jurisdictions and 30 foreign countries. Compared with test-negative respondents, test-positive respondents were more likely to report attending bars, karaoke, or nightclubs, and eating or drinking indoors near others for at least 15 min. Genomic sequencing of 20 specimens identified the SARS-CoV-2 B.1.617.2 (Delta) variant (AY.25 and AY.103 sublineages) in 15 (75%) cases, and Omicron variant (BA.1 sublineage) in five (25%) cases	[65, 66]

*PCR* polymerase chain reaction, *WGS* whole genome sequencing, *MIQ* managed isolation quarantine

## COVID-19 Mitigation and Control Measures

### Pre-travel Testing and Airport Screening Protocols

Early in the pandemic, many national guidelines included fever screening for travelers pre-departure or during transit. Some entry screening processes have also used symptom surveys and visual observations to identify potentially infected travelers. Fever scanning devices have reasonable sensitivity for detection of febrile travelers although with a relatively low positive predictive value [67]. With recognition of asymptomatic and pre-symptomatic transmission of SARS-CoV-2 and clinical studies showing that not all COVID-19 patients exhibited fevers [35, 68, 69], airport-based fever and symptom checklists are relatively low-yield screening strategies.

With the advent of more widely available diagnostic modalities including PCR and rapid antigen tests, pre-travel screening within 24 to 72 h of departure or even on-site pre-boarding rapid testing became feasible strategies to reduce the potential risk of infected travelers boarding flights or testing positive on arrival and thus requiring immediate isolation [5]. Pre-departure rapid antigen testing compared to PCR in Vancouver demonstrated the feasibility of using lateral flow, rapid antigen tests in the airport pre-departure [26]. The use of specially trained dogs to identify individuals with asymptomatic or mild COVID-19 may provide an additional innovative approach to detect infected travelers at airports, train stations, and other ports of embarkation [70, 71].

Pre-travel screening has been a frequently implemented strategy to reduce the risk of SARS-CoV-2 importation. Generally, testing was required to be conducted within 72 h before departure although from October 2021 to June 2022, the USA mandated testing within a day of travel to the USA. The yield of this approach may be relatively limited. One study of German travelers, conducted between October 2020 and January 2021, found positive pre-travel tests in only 0.58% of 521 international travelers [72]. A PCR test done 2–3 days before departure may still miss an infected traveler in the incubation period that will become infectious during travel or shortly after arrival at their destination. Examples of both have occurred. Despite pre-departure negative tests, whole genome sequencing indicated inflight transmission between Dubai and Auckland based on testing performed shortly after arrival while in managed isolation and quarantine, although the pre-departure testing had been performed 4 to 5 days before departure [50•]. Similarly, some travelers were found to have a high viral load (low cycle threshold value) on arrival in Spain despite negative pre-travel tests [54]. A modeling analysis of different strategies found that the risk of imported infection could be reduced by 80–90% through the use of testing on arrival and 7- or 14-day quarantine for test-negative individuals (relative to no testing) [73]. In summary, a variety of strategies have been utilized to attempt to reduce the importation of COVID-19 via international travelers, including controversial travel bans [74], pre-departure testing within 24 to 72 h, rapid testing immediately prior to travel, testing on arrival [75], and mandatory quarantine with testing and isolation of infected travelers. Some countries have mandated quarantine periods of as long as 21 days; these measures have resulted in unanticipated negative effects on the mental health, quality of life, and economic livelihood of travelers [19•].

Attempts to use rigorous strategies to create COVID-19-free bubbles were successfully implemented by several countries including China, Hong Kong, New Zealand, and Australia during the first year or more of the pandemic [76]. These approaches required a multi-component approach including pre-departure testing, testing on arrival, strict mandatory quarantine with testing, and isolation of infected individuals. When successfully implemented, countries like Australia and New Zealand were able to develop COVID-19-free travel corridors. However, with the advent of the highly transmissible Omicron variant of concern in late 2021 and early 2022, SARS-CoV-2 was introduced into these previously virus-free locations, leading to local spread, although often limited by intensive local testing and control measures, especially in mainland China. Ultimately, the best available strategies to reduce the spread of COVID-19 through travel need to be deployed, but with the recognition that total containment is not possible. Data support an integrated approach with pre-travel testing, ideally within 24 h of departure or airport-based screening, masking during travel, and voluntary or mandatory quarantine on arrival with testing within 5–7 days of arrival will help to mitigate the spread of COVID-19.

### Protective Efficacy of Masks and Social Distancing During Air Travel

Since recognition that SARS-CoV-2 is predominantly transmitted through respiratory droplets and small particle aerosols, face masks have become a widely used protective strategy. Observational and laboratory studies provided evidence that face masks prevent infected individuals from transmitting virus (source control) but also protect uninfected individuals from exposure to SARS-CoV-2 [77]. Similarly, a systematic review and meta-analysis found that face masks could reduce infection by as much as 85% with greater levels of protection provided by N95 or similar types of masks relative to surgical masks [78]. Good fit, mask quality, and appropriate use are all key factors influencing the degree of protection [78, 79].

While many epidemiological studies supported the protective effectiveness of masks for reducing risk of SARS-CoV-2 infection, direct evidence from airplanes, trains, and cruise ships is relatively limited. A review of in-flight transmission described several examples where strict masking policies on flights were associated with reduced or no transmission of SARS-CoV-2 in contrast to flights where masking was optional or rare [80•]. Sophisticated modeling studies have accounted for dynamic variables such as the movement of air passengers around the cabin and the variable effectiveness of masking; usage of FFP2/N95 masks reduced super-spreading events by 95–100%, while cloth masks were estimated to reduce infection rates by 40–80% [81]. The infected traveler’s overhead gasper jet and a higher backrest were mitigating factors [40]. Although prolonged masking during air travel may be uncomfortable, volunteers wearing an FFP2 protective mask showed no differences in blood oxygen saturation measurements at sea level and simulated airplane altitude (7500 ft) relative to non-mask-wearing participants [82].

Since most well-documented in-flight instances of SARS-CoV-2 transmission have involved people sitting in the same row or within two rows in front of or behind the index case [80•], reduced seating density may also help to reduce risk of in-flight transmission. In addition to masking, there is evidence from community and hospital studies that maintaining a distance between individuals reduces transmission risk with increasing protective effectiveness at 1, 2, and 3 m of distance [78]. The potential protective effect of eliminating middle seat occupancy on flights was evaluated in an experimental model, which found that this approach could lead to a reduction of transmission risk of up to 57% [83]. Unfortunately, airlines need to make use of all seats in order to maximize profits and therefore no longer use this approach.

### Protective Measures to Mitigate SARS-CoV-2 Spread on Cruise Ships

Cruise lines have had major problems with outbreaks (Table 1), which have led to careful consideration of strategies to reduce SARS-CoV-2 transmission. Many cruise ship outbreaks occurred early in the pandemic before stricter protocols were implemented by the cruise ship companies. A comprehensive analysis by the CDC of 89 voyages on 70 cruises in US water or carrying US citizens between January and April 2020 identified longer duration of travel (14- vs. 7-day voyages) as a risk factor for outbreaks [84]. In contrast, risk was lower for cruises that decreased the total number of passengers and crew members, performed testing on days 0 and 4, used daily symptom screening, isolated anyone who tested positive or developed compatible symptoms, and limited the cruise to a single port of call. Thus, a series of measures including advising full pre-cruise vaccination (ideally mandatory rather than just advised), pre-embarkation screening, daily symptom monitoring, testing on two time points shortly after departure, and effective isolation and quarantine protocols can help reduce the spread of SARS-CoV-2 on ships [85]. Data are limited on the utility of masking in this context.

## Game-Changing Therapeutic Advances

Vaccines and therapeutics were developed swiftly against SARS-CoV-2 and have played a game-changing role, especially in reopening society and restarting travel. The dazzling speed with which COVID-19 vaccines were developed and rolled out generated optimism for the world to regain normalcy [86–89]. Although some countries initially accepted only the vaccines authorized by their national health authority, many countries have come to accept vaccines that have been issued an Emergency Use Listing by the WHO [90]. Currently the most widely available, accepted, and recognized vaccines are the mRNA vaccines and adenovirus-vectored vaccines, but the list will certainly expand as more vaccines are authorized. The emergence of SARS-CoV-2 variants has moderated some expectations for normalcy due to the “immune escape” displayed by Omicron and its subvariants [91••].

Although COVID-19 vaccination programs allowed progress where vaccinated persons can assemble and can restart travel, complications arose because of vaccine hesitancy and concerns about vaccine inequality [92–95]. Misinformation and conspiracy theories regarding COVID-19 fueled doubt in science, medical experts, health authorities, and governments, and exacerbated skepticism about vaccines [92]. The exploitation of social media platforms by anti-vaccine movements and the delay in responding to misinformation led to a decline in intent to receive COVID-19 vaccines [92]. Interventions continue to be needed to increase trust in vaccines and motivation to undergo vaccination, especially with the inclusion of disadvantaged groups in mind.

Along with vaccine rollouts, documentation became a new complication for travelers due to the lack of standardized recording of COVID-19 vaccines. Each country or jurisdiction developed and utilized its own system, whether in paper or digital format. Despite the long-standing worldwide recognition of the WHO International Certificate of Vaccines or Prophylaxis (ICVP), it had not been designated as a universally accepted COVID-19 vaccine record at the start of the pandemic, and WHO’s development of digital health records had progressed slowly [7•]. The solution for the complex health passport issues includes the use of the ICVP in paper format along with digital apps. Specifically, a global framework needs to be developed for documentation of vaccination, testing type and results, and immunity [6, 7•].

The other game changer for travel is the therapeutics for ambulatory settings (Table 2). Monoclonal antibodies (currently tixagevimab-cilgavimab) for pre-exposure prophylaxis provide protection for immunocompromised travelers who may have suboptimal vaccine response, or for persons who have contraindications to COVID-19 vaccination [96••, 97]. Effective oral drugs have become available for treatment [98–101]. These oral drugs may become additional strategies for safe travel when they can serve as post-exposure prophylaxis. However, this awaits an abundant drug supply and studies supporting their use in this role.

**Table 2:** COVID-19 therapeutics with potential application to travel

**Therapeutic agent**	**Status**	**Current indication (FDA EUA, NIH)**	**Possible application for travel**	**Comments**	**References**
Tixagevimab-cilgavimab (Evusheld)	FDA, EUA	Pre-exposure prophylaxis (PrEP) for adults and adolescents (aged ≥ 12 years and weighing ≥ 40 kg) who do not have SARS-CoV-2 infection, who have not been recently exposed to an individual with SARS-CoV-2 infection, **AND** who have: •Moderate to severe immunocompromise and may have an inadequate immune response to COVID-19 vaccination; *or* •A contraindication hence unable to be fully vaccinated with COVID-19 vaccines	Pre-exposure prophylaxis for travelers at high risk of severe disease from COVID-19, who have no symptoms of COVID-19 and no confirmed exposure to COVID-19 within prior 5 days	Long-acting human monoclonal antibody that binds to the spike protein receptor-binding domain (RBD) of SARS-CoV-2 preventing binding affinity to ACE2	[100]
				PROVENT, the unpublished randomized, double-blind, placebo-controlled trial of adults > 59 years or with a pre-specified chronic medical condition or at increased risk of SARS-CoV-2 infection who had not received a COVID-19 vaccine and no history of SARS-CoV-2 infection, found a 77% reduced risk of COVID-19 compared to placebo	[96••]
Nirmatrelvir-ritonavir (Paxlovid)	FDA, EUA	Outpatient treatment of mild-moderate COVID-19 infection in patients at high risk for progressing to severe infection and possible hospitalization	Self-treatment for COVID-19: start within 5 days of symptom onset or positive viral test	A viral protease that cleaves 2 viral polyproteins leading to antiviral activity against all human coronaviruses, and packaged with ritonavir, a cytochrome P450 (CYP) 3A4 inhibitor, to boost nirmatrelvir concentrations	[98]
				EPIC-HR trial demonstrated that starting nirmatrelvir-ritonavir in adults with mild to moderate COVID-19 within 5 days of symptom onset reduced the risk of hospitalization or death through day 28 by 89% compared to placebo	
Molnupiravir	FDA, EUA	Outpatient treatment of mild-moderate COVID-19 infection in patients at high risk for progressing to severe infection and possible hospitalization (only in the case that alternative treatment options are not available or appropriate)	Self-treatment for COVID-19: start within 5 days of symptom onset or positive viral test	An oral prodrug of beta-D-N4-hydroxycytidine (NHC), a ribonucleoside that has broad antiviral activity against RNA viruses and inhibits RNA polymerase	[99]
				In the MOVe-OUT trial, molnupiravir reduced the rate of hospitalization or death by 30% compared to placebo	

*FDA* Federal Drug Administration, *EUA* emergency use authorization

## Pandemic Preparedness: Lessons for the Future

Much has been learned during the last 2 years of the COVID-19 pandemic that can serve to prepare the world for future challenges. The capacity exists to rapidly identify and sequence new viruses, and to use this knowledge to quickly develop vaccines and diagnostic tests [101, 102]. However, there is a need to be able to rapidly scale up testing access including the early development of highly sensitive and specific rapid diagnostic tests that can be used as point-of-care tests pre-travel and immediately post-travel. There needs to be recognition of the importance of specialized travel clinics in education, pre-travel testing, and provision of travel kits (e.g., rapid diagnostic tests and standby antiviral therapy). In addition, efforts to facilitate equitable distribution of effective vaccines and to ensure public trust in domestic and international responses to new pandemics will be a critical feature of future pandemic responses [102].

## Adapting to the Pandemic: the Future of Travel Medicine

Dramatic disruption of life took hold in an attempt to contain or mitigate the impact of COVID-19, with pervasive effects on travel, travel medicine, and migrant health [103, 104]. A survey by the International Society of Travel Medicine found that travel clinics faced a substantial reduction in their travel volume; the staff shifted their focus to other clinical activities (18%) or redeployed to other healthcare needs (13%), while the majority were involved with COVID-19 testing and screening (56%), providing vaccine information (53%), and vaccine administration (43%) [13•].

The COVID-19 pandemic has expedited innovation, which must continue to be incorporated into travel medicine in the future. Travel advisories and destination requirements were improved and updated weekly, such as those of the CDC Travelers’ Health Branch, UNWTO, and International Air Transport Association [105, 106]. The field of disease modeling has expanded impressively and provided estimates on many aspects of COVID-19. Molecular epidemiology through genome sequencing has accelerated rapidly. Worldwide, educational programs were modified to follow public health advice. Leading travel medicine educational programs transformed to successfully deliver high-quality online webinars and courses, online examinations, and an international conference using an immersive virtual reality platform [12]. Collaborators also met regularly online, overcoming the previous barrier imposed by distance and cost.

The future of travel medicine is robust. Travel-related health risks persist and concern for non-infectious diseases has increased [10, 11]. COVID-19 has raised public awareness about the interaction of travel with cross-border infections and emerging infections, and again illustrated the role of the traveler as sentinel of infectious diseases [107]. It has also highlighted the vulnerability of the traveler with chronic medical illnesses [108•]. COVID-19 is likely to remain as an endemic infection, and surveillance will be as important as prevention. COVID-19 gave an impetus to advance technology for virtual appointments, to adopt mobile devices and GPS in contact tracing, and to use digital applications in research and surveillance of other travel-related infections such as arboviral diseases and rabies [17, 109, 110]. The future of travel medicine should harness these new platforms in its clinical work, research, and education.

## Strengths and Limitations of Review

The volume of new publications relating to COVID-19 continues to increase rapidly, making it necessary to update reviews on this subject at regular intervals. An effort has been made to draw on the most recently published sources relevant to COVID-19 and international travel in this review. Our search strategy did not include specialized social science or psychology sources, which may have contained relevant material. Our literature search was restricted to articles published in the English language, and potentially relevant studies published in other languages may therefore have been overlooked.

## Conclusions

The ongoing COVID-19 pandemic has had a profound impact on the travel industry and on the experiences of the travelling public, who are now familiar with the requirements of COVID-19 vaccine certification, pre- and post-departure diagnostic testing, rapid antigen self-testing, mask wearing, and the critical importance of comprehensive travel insurance. While novel vaccines and antiviral agents have greatly reduced the risk of severe hospitalization and death, the threat of further variants that show vaccine or immune escape looms large and complicates efforts to return to a normal travel environment. Travel medicine providers have demonstrated resilience in adapting to the pandemic. Travel medicine should leverage the technological and behavioral progress of the pandemic in insulating itself against future global shocks. Future research should assess the long-term impact of the pandemic on traveler health behavior and on the practice of travel medicine.
